# Clinical effect of flunarizine combined with duloxetine in the treatment of chronic migraine comorbidity of depression and anxiety disorder

**DOI:** 10.1002/brb3.2689

**Published:** 2022-07-05

**Authors:** Junliang Zhu, Jianhuang Chen, Kaixue Zhang

**Affiliations:** ^1^ Department of Neurology Liuyang Jili Hospital Changsha Hunan China

**Keywords:** chronic migraine, comorbid depression and anxiety disorders, duloxetine, flunarizine, inflammatory reaction

## Abstract

**Background:**

Migraine is common in primary headaches, and with the development of social economy and the increase in living pressure, the prevalence of migraine has an upward trend.

**Objective:**

To observe the clinical effect of flunarizine combined with duloxetine in the treatment of chronic migraine with comorbid depression and anxiety disorders and to provide a reference for clinical treatment.

**Methods:**

A total of 118 patients with chronic migraine complicated with depression and anxiety disorder admitted to our hospital from June 2018 to August 2020 were selected and divided into two groups according to treatment methods, 59 cases in each group. The control group was treated with flunarizine combined with loxoprofen sodium, and the observation group was treated with flunarizine combined with duloxetine. The changes of electroneurophysiological indexes, tumor necrosis factor‐α (TNF‐α), interleukin‐6 (IL‐6), high sensitivity‐C reactive protein (hs‐CRP), Hamilton depression scale (HAMD) score, and Hamilton anxiety scale (HAMA) score before and after treatment in the two groups were recorded, and the total effective rate of clinical treatment in the two groups was counted.

**Results:**

After treatment, TNF‐α, IL‐6, and hs‐CRP in the two groups decreased gradually (*p* < .05). Further comparison between groups showed that TNF‐α, IL‐6, and hs‐CRP in the observation group were lower than those in the control group (*p* < .05). After treatment, the HAMD score and the HAMA score of the two groups decreased gradually (*p* < .05). Further comparison between the two groups showed that HAMD score and HAMA score of the observation group were lower than those of the control group (*p* < .05).

**Conclusion:**

Flunarizine combined with duloxetine in the treatment of chronic migraine with depression and anxiety disorder can effectively improve neuroelectrophysiological indexes, reduce inflammation, and reduce depression and anxiety.

## Graphical abstract

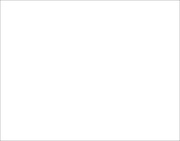

## 1 INTRODUCTION

Chronic migraine is a common type of primary headache in clinic, with recurrent moderate to severe head pain on one or both sides as the main symptom, which is often accompanied by autonomic nervous symptoms such as nausea, vomiting, and phobia. Improper diet, sound and light stimulation, overwork, and negative emotions can cause or aggravate headache. Chronic migraine is associated with anxiety and depression (Bhering Martins et al., 2020). Physical therapy and drug therapy are common methods of clinical treatment of this disease. Flunarizine is the calcium antagonist of piperidine difluoride derivative, which has a certain therapeutic effect on epilepsy and migraine. But it cannot reduce negative emotions and is not conducive to the control of the disease (Peres et al., 2019; Wang et al., 2022).

Duloxetine is a selective serotonin and norepinephrine reuptake inhibitor, which is often used in the treatment of severe depression, diabetic peripheral neuralgia, and female stress urinary incontinence (Gonzalez‐Martinez et al., 2020). Studies have found that duloxetine hydrochloride enteric‐coated capsules can effectively relieve anxiety and depression, reduce the frequency of migraine attacks and pain, and improve the quality of life of patients (Liu et al., 2021; Peres et al., 2019). However, there are few studies on its effects on neuroelectrophysiological indexes and inflammatory indexes. This study observed the clinical effect of flunarizine combined with duloxetine in the treatment of chronic migraine with depression and anxiety disorder, and provided a reference for clinical treatment.

## 2 MATERIALS AND METHODS

### 2.1 General information

A total of 118 patients with chronic migraine complicated with depression and anxiety disorder admitted to our hospital from June 2018 to August 2020 were selected and divided into two groups according to treatment methods, 59 cases in each group. Thirty‐one males and 28 females in the control group; aged 19−75 years old, with an average of 51.66 ± 11.89 years old; the course of disease was 1−18 years, with an average of 8.63 ± 2.77 years. There were 22 cases of migraine without aura and 38 cases of migraine with aura. Thirty‐four males and 25 females in the observation group; aged 20−75 years old, with an average of (50.53 ± 12.84) years old; course of disease was 1−18 years, with an average of 8.19±2.93 years. There were 26 cases of migraine without aura and 34 cases of migraine with aura. The general data was compared between the two groups, and the difference was not significant (*p* > .05). Inclusion criteria: (1) chronic migraine meets the criteria of “expert consensus on migraine diagnosis and prevention” (Eigenbrodt et al., 2021). (2) Depression and anxiety disorders meet the diagnostic criteria in the “Chinese Classification and Diagnostic Criteria for Mental Disorders (CCMD‐3)” (Chen, 2002). (3) Age ≥ 18 years old, ≤ 75 years old. (4) Mentally normal, with treatment and efficacy evaluation. (5) Complete clinical data. Exclusion criteria: (1) Those with history of alcohol abuse, sedatives, and analgesics. (2) Hamilton Depression Scale (HAMD) score < 8, Hamilton Anxiety Scale (HAMA) score < 7. (3) Patients with malignant tumors, systemic infections, hematopoietic, and immune system diseases. (4) Secondary headache caused by other diseases. (5) Women in pregnancy or lactation. (6) Allergic constitution.

### 2.2 Methods

The control group was treated with flunarizine hydrochloride capsules (Xi'an Yangsen Pharmaceutical Co., Ltd, specification: 5 mg, Chinese medicine standard H10930003) combined with loxoprofen sodium tablets (Shanghai Xudong Haipu Pharmaceutical Co., Ltd, specification: 60 mg, Chinese medicine standard H20030614), and oral flunarizine 5−10 mg/time, 1 time/day; loxoprofen sodium 60 mg/time, 3 times/day.

The observation group was treated with flunarizine combined with duloxetine hydrochloride enteric‐coated tablets (Shanghai Sino‐Western Pharmaceutical Co., Ltd, specification: 20 mg, Chinese medicine standard word H20061261). The use and dosage of flunarizine were the same as those of the control group, and duloxetine hydrochloride was taken orally, 60 mg/time, 1 time/day. Both groups were treated for 8 weeks.

### 2.3 Observation indicators and detection methods

The changes of neuroelectrophysiological indexes, tumor necrosis factor‐α (TNF‐α), interleukin‐6 (IL‐6), high‐sensitivity C‐reactive protein (hs‐CRP), HAMD score, and HAMA score before and after treatment in the two groups were recorded, and the total effective rate of clinical treatment in the two groups was counted.

HAMD score and HAMA score: depression and anxiety of patients were evaluated. The higher the score, the heavier the negative emotion. HAMD score < 8 points means no depression; ≥8 points means possible depression; ≥20 points means definitely depression; ≥35 points means severe depression. HAMA score < 7 points means no anxiety; ≥7 points means possible anxiety; ≥14 points means definitely anxiety; ≥ 21 points means obvious anxiety; ≥ 29 points means severe anxiety (Thompson, 2015).

The auditory oddball task was performed before and after 8 weeks of treatment. The standard stimulation was 1000 Hz, 90 dB and the duration was 160 ms. Target stimulation: 1800 Hz, 90 dB, and duration: 160 ms. The amplitude and latency of N2, P3a, and P3b were recorded.

Before treatment and 8 weeks after treatment, 3 ml fasting venous blood was extracted from patients and centrifuged at 4000 r/min for 10 min. Serum levels of TNF‐α, IL‐6, and hs‐CRP were detected by enzyme‐linked immunosorbent assay. Detection instrument: Hyperion MR III enzyme labeled instrument, kit for Nanjing Jiancheng Institute of Bioengineering products.

### 2.4 Efficacy criteria

Clinical efficacy criteria: (1) Recovery: after treatment, the clinical symptoms of migraine disappeared, imaging (Transcranial Doppler) showed normal or basically normal. (2) Significantly effective: after treatment, the clinical symptoms of migraine were significantly reduced compared with those before treatment, the frequency was reduced by ≥ 90%, and imaging showed significant improvement. (3) Effective: after treatment, the clinical symptoms of migraine were alleviated compared with those before treatment, and the frequency of 50% ≤ attack was reduced by < 90%, and the imaging findings were improved. (4) Invalid: did not meet the above standards. Total effective rate = cure rate + significant efficiency + effective rate.

### 2.5 Statistical methods

SPSS19.0 was used to process the data. The measurement index was described by (¯χ ± s). The t test was used for the comparative analysis of the data. The number of cases was used for the enumeration data. The Chi‐square test was used for the comparative analysis of the data. *p* < .05 indicated that the difference was significant.

## 3 RESULTS

### 3.1 Comparison of curative effect between the two groups of patients

The total effective rate of the observation group (91.53%) was higher than that of the control group (77.97%), and the difference was significant (*p* < .05) (see Table 1).

**TABLE 1:** Comparison of curative effects between two groups (*n* [%])

Group	Number of cases	Recovery	Remarkable effect	Effective	Invalid	Total effective rate
Control group	59	14 (23.73)	17 (28.81)	15 (25.42)	13 (22.03)	46 (77.97)
Observation group	59	19 (32.20)	21 (35.59)	14 (23.73)	5 (8.47)	54 (91.53)
*χ* ^2^						4.196
*p*						.041

### 3.2 Comparison of neurophysiological indicators between the two groups of patients

The two groups were compared before treatment, and the differences were not significant (*p* > .05). After treatment, the amplitudes of N2, P3a, and P3b in the two groups increased gradually (*p* < .05), and the latency of N2, P3a, and P3b decreased gradually (*p* < .05). Further comparison between the two groups showed that the amplitudes of N2 (Table 2), P3a (Table 3), and P3b (Table 4) in the observation group were higher than those in the control group (*p* < .05). The incubation periods of N2 and P3a were lower than those the control group (*p* < .05), but the latency of P3b was not significantly different from that of the control group (*p* > .05) (see Table 2).

**TABLE 2:** Comparison of neuroelectrophysiological indexes (N2) between the two groups (χ ± s)

Group	Number of cases	N2			
		Amplitude (μV)		Incubation period (ms)	
		Before treatment	After treatment	Before treatment	After treatment
Control group	59	3.26 ± 1.02	5.21 ± 1.23^*^	201.32 ± 25.33	190.23 ± 21.66^*^
Observation group	59	3.31 ± 1.14	7.52 ± 1.33^*^	197.23 ± 30.26	182.36 ± 20.75^*^
*t*		0.251	9.794	0.796	2.015
*p*		.802	.000	.428	.046

*Note*: Compared with before treatment,^*^ *p* < .05.

**TABLE 3:** Comparison of neuroelectrophysiological indexes (P3a) between the two groups (¯χ ± s)

Group	Number of cases	P3a			
		Amplitude (μV)		Incubation period (ms)	
		Before treatment	After treatment	Before treatment	After treatment
Control group	59	10.25 ± 3.52	13.02 ± 3.44^*^	271.56 ± 25.96	262.06 ± 24.77^*^
Observation group	59	10.16 ± 3.68	14.75 ± 3.06^*^	268.33 ± 29.14	251.36 ± 30.15^*^
*t*		0.136	2.886	0.636	2.106
*p*		.892	.005	.526	.037

*Note*: Compared with before treatment,^*^ *p* < .05.

**TABLE 4:** Comparison of neuroelectrophysiological indexes (P3b) between the two groups (¯χ ± s)

Group	Number of cases	P3b			
		Amplitude (μV)		Incubation period (ms)	
		Before treatment	After treatment	Before treatment	After treatment
Control group	59	6.56 ± 1.32	7.68 ± 1.31^*^	312.02 ± 25.85	291.25 ± 21.42^*^
Observation group	59	6.49 ± 1.38	8.21 ± 1.24^*^	309.68 ± 31.22	288.96 ± 23.63^*^
*t*		0.282	2.257	0.443	0.552
*p*		.779	.026	.658	.582

*Note*: Compared with before treatment,^*^ *p *< .05.

### 3.3 Comparison of TNF‐α, IL‐6, and hs‐CRP levels between the two groups

The two groups were compared before treatment, and the difference was not significant (*p* > .05). After treatment, TNF‐α, IL‐6, and hs‐CRP in the two groups decreased gradually (*p* < .05). Further comparison between the two groups showed that TNF‐α, IL‐6, and hs‐CRP in the observation group were lower than those in the control group (see Table 5).

**TABLE 5:** TNF‐α in two groups, Comparison of IL‐6 and hs CRP levels (¯χ ± s)

		TNF‐α (pg/ml)		IL‐6 (pg/ml)		hs‐CRP (mg/l)	
Group	Number of cases	Before treatment	After treatment	Before treatment	After treatment	Before treatment	After treatment
Control group	59	10.52 ± 2.36	7.12 ± 1.96^*^	9.12 ± 2.89	5.85 ± 1.36^*^	6.86 ± 2.12	4.02 ± 1.14^*^
Observation group	59	11.01 ± 2.27	6.38 ± 1.44^*^	9.20 ± 2.76	5.03 ± 1.21^*^	6.79 ± 2.22	3.26 ± 0.82^*^
*t*		0.149	2.337	0.154	3.460	0.175	4.157
*p*		.253	.021	.878	.001	.861	.000

*Note*: Compared with before treatment,^*^ *p *< .05.

### 3.4 Comparison of HAMD score and HAMA score between the two groups

The two groups were compared before treatment, and the difference was not significant (*p* > .05). After treatment, the HAMD score and HAMA score of the two groups decreased gradually (*p* < .05). Further comparison between the two groups showed that the HAMD score and HAMA score of the observation group were lower than those of the control group (*p* < .05) (see Table 6).

**TABLE 6:** Comparison of HAMD score and HAMA score between the two groups (¯χ ± s, branch)

		HAMD score		HAMAscore	
Group	Number of cases	Before treatment	After treatment	Before treatment	After treatment
Control group	59	20.23 ± 4.85	14.36 ± 3.96^*^	16.85 ± 4.07	11.23 ± 3.63^*^
Observation group	59	21.02 ± 5.11	9.21 ± 3.02^*^	17.04 ± 4.23	7.02 ± 3.11^*^
*t*		0.861	7.943	0.249	6.765
*p*		.391	.000	.804	.000

*Note*: Compared with before treatment,^*^ *p *< .05.

## 4 DISCUSSION

Migraine is a type of abnormal vascular function disease of the head and face caused by dysregulation of the nerve. It occurs for the first time in childhood and adolescence and reaches its peak in middle age and young people. It often has genetic background, which can seriously affect the study and life of patients (Gupta et al., 2008). At present, the pathogenesis of migraine is not fully understood. Previous studies have shown that it is closely related to increased brain excitability, abnormal ion channels, extended cortical inhibition, and abnormal central neurotransmitter levels. Excitatory amino acid and dopamine receptor gene mutations are important mechanisms of migraine (Pozo‐Rosich et al., 2021). Migraine and anxiety and depression have a common biochemical basis, often accompanied by complications (Lardreau, 2012). Flunarizine is a vasodilator that selectively blocks calcium and voltage‐dependent sodium channels to relieve pain and reduce the number of episodes of periodic pain (Pozo‐Rosich & Torres‐Ferrus, 2020). Loxoprofen sodium is a nonsteroidal anti‐inflammatory drug widely used in the treatment of rheumatoid arthritis, low back and leg pain, neck shoulder wrist syndrome, migraine, and other painful diseases (Lisicki & Schoenen, 2020).

Because anxiety and depression can promote migraine, it is necessary to relieve negative emotions in the treatment of chronic migraine with depression and anxiety disorder (Cayir et al., 2020). Duloxetine has dual effects of antidepression and central analgesia and has good effect on negative emotions such as anxiety and depression and their induced somatic complications (Kim et al., 2019). This study found that the total effective rate of flunarizine combined with duloxetine was higher than that of flunarizine combined with loxoprofen sodium. After treatment, the HAMD and HAMA scores were lower than those treated with flavors combined with loopholes sodium. The results suggest that flunarizine combined with duloxetine in the treatment of chronic migraine with depression and anxiety disorder comorbidity can better control migraine attacks and reduce depression and anxiety. This is consistent with the existing clinical findings (Aljunaid et al., 2020). This is because the decrease in serotonin, norepinephrine, and other neurotransmitter levels is closely related to pain and emotional disorders. Duloxetine has a high affinity to 5‐hydroxytryptamine and norepinephrine receptors, and can inhibit the reuptake of 5‐hydroxytryptamine and norepinephrine, thereby increasing the levels of neurotransmitters such as 5‐hydroxytryptamine and norepinephrine in the central nervous system, which is beneficial to the control of migraine and the improvement of mood.

N2 is the response inhibition wave, reflecting the time from receiving stimulation to response execution. P3a originates from the temporal parietal cortex, reflecting automatic attention; P3b originates from the hippocampus, reflecting active attention (Hashimoto et al., 2019). This study found that the amplitude of N2, P3a, and P3b in patients treated with flunarizine combined with duloxetine was higher than that in patients treated with flunarizine combined with loxoprofen sodium, and the latency of N2 and P3a was lower than that in patients treated with flunarizine combined with loxoprofen sodium, but there was no significant difference in the latency of P3b between the two groups. This result suggests that flunarizine combined with duloxetine in the treatment of chronic migraine with comorbid depression and anxiety disorders can effectively improve the neurological function of patients. This is because duloxetine can activate 5‐hydroxytryptamine and norepinephrine pathways, increase the release of brain‐derived neurotrophic factors, restore neural plasticity, and promote nerve injury repair.

TNF‐α is an early factor of inflammatory response, which can promote the release of proinflammatory factors such as IL‐6 and further aggravate the inflammatory response (Guliyeva et al., 2020). hs‐CRP is a sensitive inflammatory marker, which increases rapidly in trauma, inflammation, and infection (Hagen et al., 2020). The body of migraine patients was in a state of microinflammation, and the serum levels of TNF‐α, IL‐6, and hs‐CRP were at a high level (Taheri et al., 2021). This study found that TNF‐α, IL‐6, and hs‐CRP in patients treated with flunarizine combined with duloxetine were lower than those in patients treated with flunarizine combined with loxoprofen sodium. This result suggests that flunarizine combined with duloxetine in the treatment of chronic migraine with comorbid depression and anxiety disorders can reduce the inflammatory response.

In summary, flunarizine combined with duloxetine in the treatment of chronic migraine with depression and anxiety disorder comorbidity can effectively improve neuroelectrophysiological indexes, reduce inflammation, and alleviate depression and anxiety.

## CONFLICT OF INTEREST

The author(s) declared no potential conflicts of interest with respect to the research, authorship, and/or publication of this article.

### PEER REVIEW

The peer review history for this article is available at https://publons.com/publon/10.1002/brb3.2689

## Data Availability

The datasets generated and analyzed during the current study are available from the corresponding author on reasonable request.
